# Supplementary material for: Vaterite/Fucoidan Hybrid Microparticles: Fabrication, Loading of Lactoferrin, Structural Characteristics and Functional Properties
Source: Mar Drugs. 2025 Nov 5;23(11):428. doi: 10.3390/md23110428 (PMC12653817; doi:10.3390/md23110428)
Supplement: Supplementary file 1 [file marinedrugs-23-00428-s001.zip › marinedrugs-3933275-supplementary.pdf]

Supplementary Materials

1. Supplementary data of EDS

A (CC)

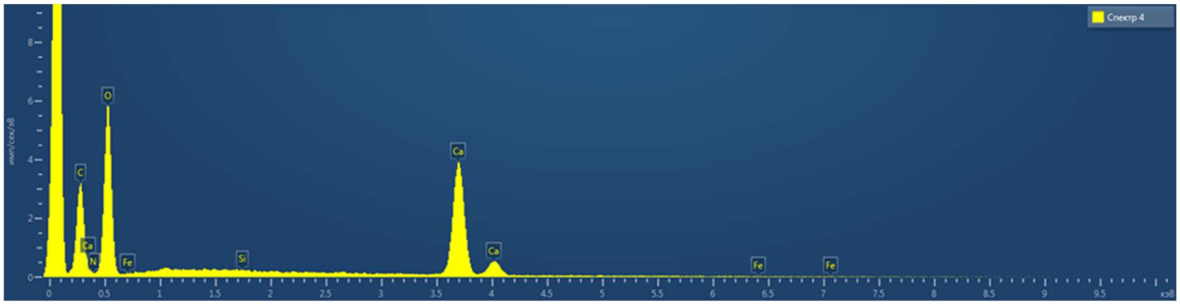

B (CCF)

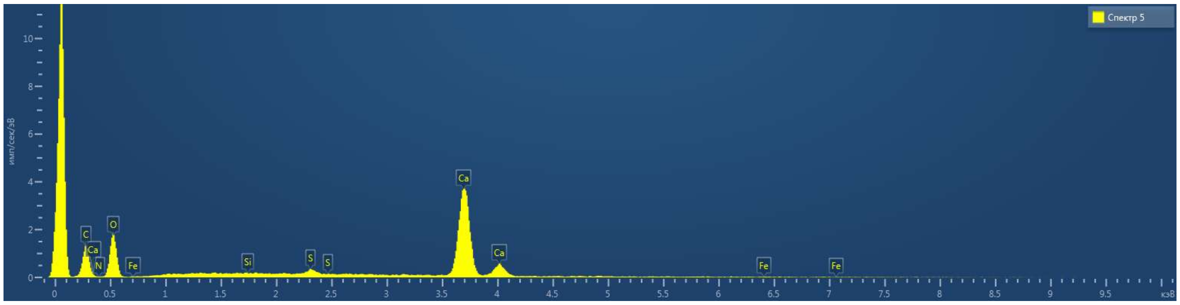

C (CCFL)

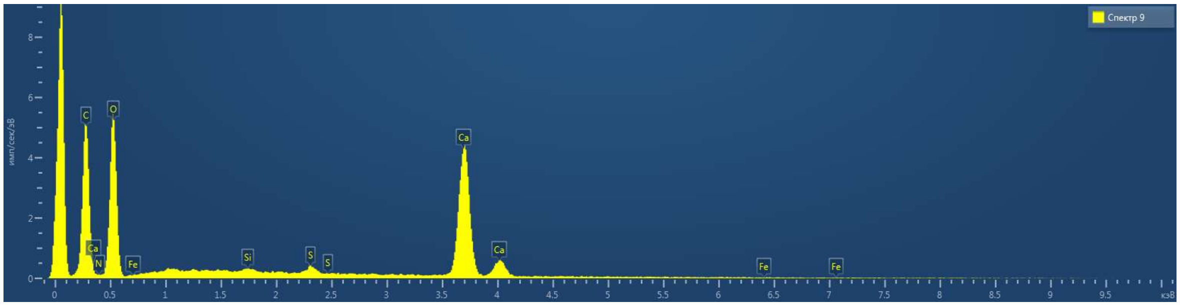

Figure S1. EDS spectrum of microparticles CC (A), CCF (B), CCFL (C).

Table S1. The content of elements in preparations according to EDS data.

Element	Miicroparticles		
	CC	CCF	CCFL
	Atom. %		
C	**20.22**	**16.79**	**30.90**
N	0.00	0.00	0.00
O	**57.93**	**42.99**	**47.01**
Si	0.00	0.00	0.23
S	**0.00**	**1.05**	**0.70**
Ca	**21.85**	**39.17**	**21.22**
Fe	0.00	0.00	0.00
Sum	100.00	100.00	100.00

2. Supplementary data of TGA

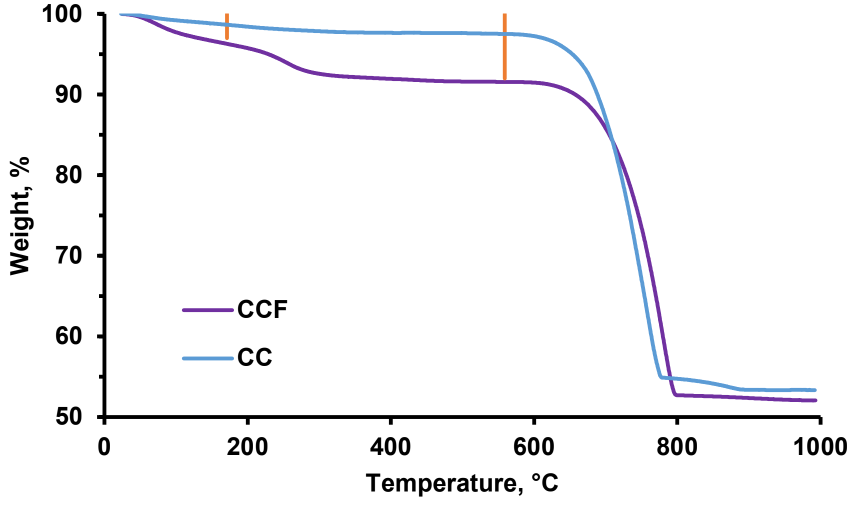

Figure S2. TGA curve showing the change in mass of vaterite microparticle samples.

3. The colorimetric method for determination of sugars (Dubois Assay)

The standard fucoidan concentrations 0 - 0.35 mg mL^{-1} were used for calibration of the method (Figure S2 (A)) by Dubois assay as follows: 0.15 mL fucoidan solution or negative control was mixed with 0.15 mL 5% phenol and 0.75 mL concentrated sulfuric acid. The mixture was vortexed and after 30 min, the absorbance at 485 nm was read. Microparticles CCF or CCFL were first resuspended in double-distilled water (1 mg mL^{-1}) and then 0.15 mL suspension was mixed with other reagents as described above.

As Figure S2 (B) shows, oligosaccharides in glycoprotein lactoferrin also can be determined by Dubois assay but 0 – 0.5 mg mL^{-1} lactoferrin absorbance was 6 times less than that of fucoidan.

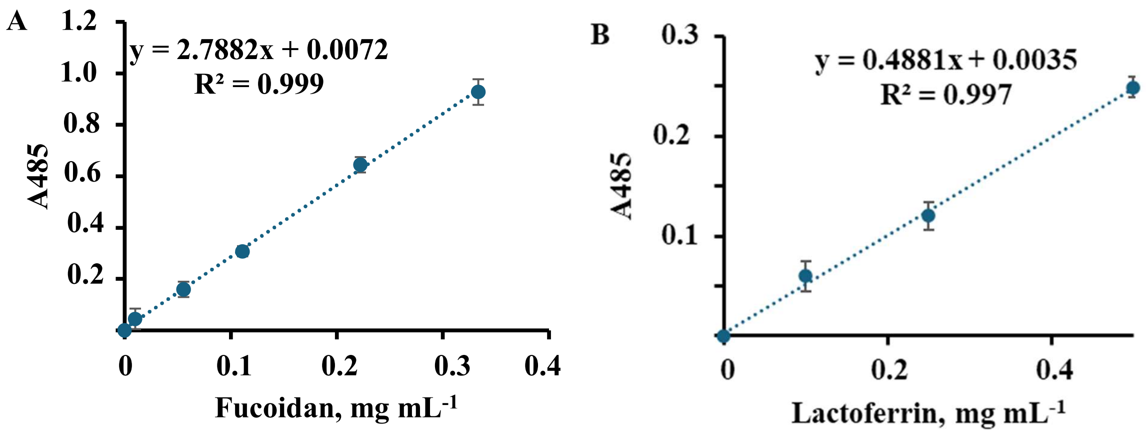

Figure S3. Calibration dependence for concentration of fucoidan (A) and lactoferrin (B) determined by Dubois assay.

4. Lowry's protein assay

The following reagents were prepared: solution A was 2% (w/v) sodium carbonate in 0.1 M sodium hydroxide; solution B was 0.781 g $CuSO_4 \cdot 5H_2O$ and 1.3837 g sodium citrate dissolved in 100 mL H_2O. Solution C was the mixture of 50 volume of solution A and 1 volume of solution B prepared before determination. Folin-Ciocalteau reagent was diluted to 1M acid according to the supplier's instruction.

To 0.2 mL of the 0-0.5 mg mL^{-1} lactoferrin solution, 1mL of solution C was added, mixed thoroughly by vortexing and was left at room temperature for 10 min. Then 0.1 mL of diluted Folin-Ciocalteau reagent was added, mixed rapidly, and incubated for 30 min at room temp. Absorbance at 750 nm was measured against reagent blank not containing protein. The calibration dependence (Figure S3) was used to determine lactoferrin concentration in the experimental samples.

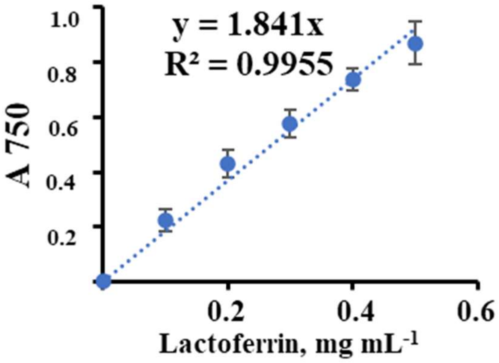

Figure S4. Calibration dependence for determination of lactoferrin by Lowry's method.

The lactoferrin incorporation into the microparticles was calculated from the difference between its concentration in the initial solution and in supernatants and washing solutions at particles fabrication. To determine protein in the microparticles, they were previously dissolved in 15% HCl.

5. X-ray assay

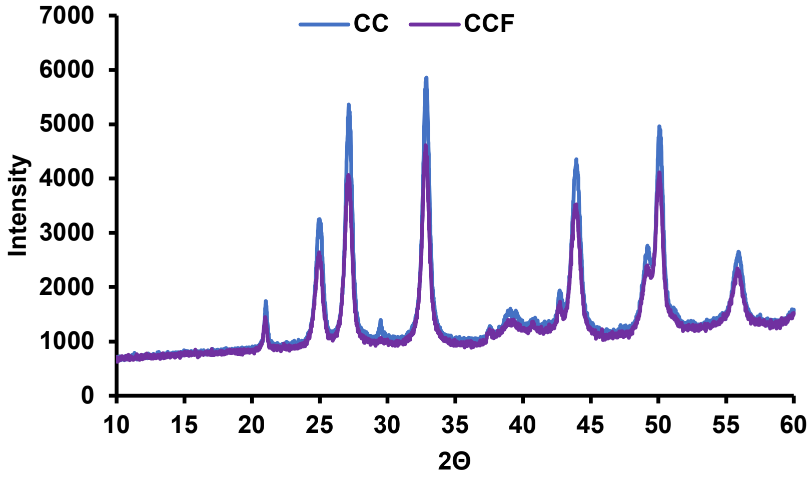

Figure S5. X-ray phase diagrams of vaterite microparticles.
